# Gastric electrical stimulation treatment of type 2 diabetes: effects of implantation versus meal-mediated stimulation. A randomized blinded cross-over trial

**DOI:** 10.14814/phy2.12456

**Published:** 2015-07-14

**Authors:** Harold E Lebovitz, Bernhard Ludvik, Jaroslaw Kozakowski, Wieslaw Tarnowski, Mateusz Zelewski, Irit Yaniv, Tse’ela Schwartz

**Affiliations:** 1 State University of New York Health Science Center at BrooklynBrooklyn, New York; 2 Internal Medicine, Medical University of ViennaVienna, Austria; 3 Center of Postgraduate Medical Education, Orlowski HospitalWarsaw, Poland; 4 Medical Center of Postgraduate Education, Bielanski HospitalWarsaw, Poland; 5 MetaCure Ltd.Orangeburg, New York; 6 Research Division at GCP Clinical StudiesTel Aviv, Israel

**Keywords:** Gastric electrical stimulation, glycemic control, gut-brain axis, hemoglobin A1c, placebo effect

## Abstract

Gastric electrical stimulation with the implanted DIAMOND device has been shown to improve glycemic control and decrease weight and systolic blood pressure in patients with type 2 diabetes inadequately controlled with oral antidiabetic agents. The objective of this study was to determine if device implantation alone (placebo effect) contributes to the long-term metabolic benefits of DIAMOND® meal-mediated gastric electrical stimulation in patients with type 2 diabetes. The study was a 48 week randomized, blinded, cross-over trial in university centers comparing glycemic improvement of DIAMOND® implanted patients with type 2 diabetic with no activation of the electrical stimulation (placebo) versus meal-mediated activation of the electrical signal. The endpoint was improvement in glycemic control (HbA1c) from baseline to 24 and 48 weeks. In period 1 (0–24 weeks), equal improvement in HbA1c occurred independent of whether the meal-mediated electrical stimulation was turned on or left off (HbA1c −0.80% and −0.85% [−8.8 and −9.0 mmol/mol]). The device placebo improvement proved to be transient as it was lost in period 2 (25–48 weeks). With electrical stimulation turned off, HbA1c returned toward baseline values (8.06 compared to 8.32%; 64.2 to 67.4 mmol/mol, *P* = 0.465). In contrast, turning the electrical stimulation on in period 2 sustained the decrease in HbA1c from baseline (−0.93%, −10.1mmol/mol, *P* = 0.001) observed in period 1. The results indicate that implantation of the DIAMOND device causes a transient improvement in HbA1c which is not sustained beyond 24 weeks. Meal-mediated electrical stimulation accounts for the significant improvement in HbA1c beyond 24 weeks.

## Introduction

Neural electrical signaling plays a major role in regulating metabolic processes. The application of external nonexcitatory electrical signals can be used to modify disturbed physiology in many disease states such as neuromuscular abnormalities, myocardial rhythm disturbances, obesity, and diabetes mellitus (Behar 2014). The application of external electrical stimulation requires a device to deliver the specific applied signal. Clinical trials with electrical-stimulating devices are fraught with difficulties and proof of significant meaningful clinical results require demonstration of positive results in a randomized, blinded, placebo-controlled clinical trial. In the evaluation of the effects obtained, it is necessary to determine the contribution of the device implantation itself, if any, and the contribution due to the specific electrical signal delivered by the device. With device implantation, a potential beneficial placebo effect could be substantial and of modest duration. Thus, trial comparisons with active device treatment need be of adequate duration to differentiate active device-related from placebo-related effects.

Gastric electrical stimulation delivered by the DIAMOND® device [Metacure, Orangeburg, NY] has been shown in previous studies to improve glycemic control, reduce body weight, and lower systolic blood pressure in patients with type 2 diabetes inadequately controlled with oral antidiabetic agents (Sanmiguel et al. 2007, 2009; Lebovitz et al. 2013, 2015). The gastric electrical stimulation increases the force of antral contractions and activates afferent neural receptors to hindbrain nuclei which are thought to regulate satiety, blood pressure, and hepatic glucose production and islet hormone secretions (Peles et al. 2003; Lebovitz et al. 2015). A fundamental question with the DIAMOND® implantable device as with all electrical stimulatory devices is whether there is a placebo effect due to the implantation of the device itself and, if so, what is its magnitude and how long does it persist. Such an effect could be due to the implanted electrodes transiently modifying and activating the normal antral and adjacent tissue neural network. This study was carried out to determine whether DIAMOND ® implantation has placebo effects and if so, how much of its effects are related to the placebo effect and how much to the electrical impulses generated by the pulse generator during meal stimulation. This report presents the results of a randomized, controlled, blinded clinical trial designed to answer these questions.

## Materials and Methods

### Device

The DIAMOND® device (Metacure) has been described elsewhere (Lebovitz et al. 2013). The device consists of three pairs of bipolar electrodes, a pulse generator, an external battery charger and an external programmer. The three bipolar electrodes are attached to the gastric fundus, anterior antral area, and posterior antral area by laparoscopic surgery. The electrodes are attached to a pulse generator which is placed in a surgically constructed pocket in the abdominal subcutaneous fat. The fundal electrode detects nutrient ingestion and relays a signal to the pulse generator. The pulse generator sends impulses to the two antral regions causing increased force of antral contractions and stimulating local neural endings which transmit signals from the gut to the hindbrain presumably to the nucleus tractus solitaries (Peles et al. 2003). An additional action of the electrical stimulation is to activate the antral region on detection of food ingestion rather than 30 or more minutes delay as required for the food to reach the antral regions (Sanmiguel et al. 2007). Clinical data suggest the gut-hindbrain impulses are forwarded to the hypothalamus where they are interpreted and integrated with other signals and generate responses which are relayed back through the more caudal brainstem to mediators of glycemic control (liver and pancreatic islets) (Lebovitz et al. 2015; Wong et al. 2015).

### Patients and protocol design

**Figure 1:**
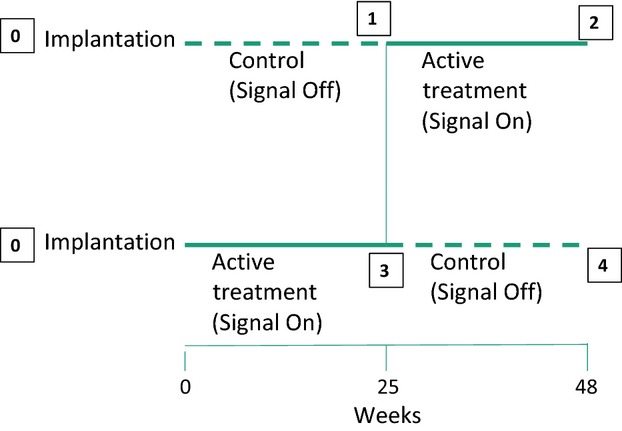
Design of the cross-over study. All patients were implanted at time 0. At the end of week 1, patients were randomized to have the electrical impulse signal turned on (ACTIVE TREATMENT) or left off (CONTROL). At 25 weeks the ACTIVE TREATMENT group had the electrical signal turned off (CONTROL) and the CONTROL group had the electrical signal turned on (ACTIVE TREATMENT). Boxes 1 and 4 are completion of the electrical impulse signal off. Boxes 2 and 3 are the conclusions of the electrical impulse signal on.

Patients with type 2 diabetes inadequately controlled on oral antihyperglycemic agents were screened, had routine laboratory measurements and were implanted with the DIAMOND® device. All patients signed informed consent; the protocol was approved by the institutional review boards of each research center; and the patients were treated appropriately as defined by the guidelines for the treatment of research subjects. The studies were performed by the co-authors at The Medical University of Vienna, Orlowski Hospital Warsaw, Poland and Bielanski Hospital Warsaw, Poland. The patients had to have been on a stable treatment regimen for a minimum of 3 months. Patients were >21 years of age (mean 52 ± 8 years), 29 women and 22 men, and had HbA1c >7.5% (58 mmol/mol) and ≤10.5% (91 mmol/mol). The patients were told to maintain their ordinary diabetic diet. A total of 51 patients were implanted. One week following implantation, the patients were randomized in a blinded fashion to either no impulse generation from week 1 through week 24 (CONTROL) or meal-mediated impulse generation (ACTIVE TREATMENT). At week 25, the groups were cross-over and received the other treatment paradigm from week 25 through 48. Figure1 is a schematic of the protocol design. A 48 week cross-over design was the preferred design as the study recruited patients with inadequate baseline glycemic control and regulatory agencies and institutional review boards limit placebo treatment in such patients to 6 months.

### Laboratory measurements

Fasting plasma triglycerides, HbA1c, fasting plasma glucose, and body weight were measured at baseline and 6 week intervals. The data analyses included the baseline, the midpoint and the conclusion of each period (0, 12, 24, 36, and 48 weeks). The HbA1c values are reported as the percent of total hemoglobin that is glycosylated as well as the International Federation of Clinical Chemistry and Laboratory Medicine (IFCC) reference method which reports it as mmol/mol (ADA et al. 2007).

### Statistical analysis

We analyzed the data by several different approaches including the AB/BA cross-over model as described by Stephen Senn (Senn 2002). In that model, there are three types of effects: treatment, period, and carry over. The Satterthwaite correction was used to correct for the unequal variance (Satterthwaite 1946). The carry over effect was not significant and the treatment effect was adjusted for the period effect. This analysis yields the half period differences.

The data were also analyzed as a parallel trial starting from either baseline (time 0) or 24 weeks with the outcome being the difference in A1C between 0 and 48 weeks or between 24 and 48 weeks. This method analyzes the period differences.

## Results

Of the 51 patients implanted, valid data were available for analysis in 43 patients; 19 who were ACTIVE TREATMENT in period 1 and 24 who were CONTROL in period 1. Three patients (two rescued with insulin and one because of personal reasons) had valid data through weeks 40 or 44 and their data were carried forward by intention to treat and included in the analysis. Of the eight patients dropping out of the study, one patient died of a massive myocardial infarction unrelated to the treatment shortly after completion of period 1 (CONTROL), one suffered a cerebrovascular accident early in period 1 (CONTROL), one patient’s A1C values were uninterpretable because of laboratory error and the other five withdrew from the study within the first period for personal reasons (two CONTROL and three ACTIVE TREATMENT).

Table1 provides the mean baseline HbA1c, fasting plasma glucose; weight, fasting plasma triglycerides, plasma cholesterol, and blood pressure for the patients randomized to ACTIVE TREATMENT or CONTROL treatment in the first period. There were no statistically significant differences in these parameters between the two randomized groups.

**Table 1:** Baseline characteristics of 43 patients completing the 48 week cross-over study

Characteristic	ACTIVE (week 1–24) CONTROL (week 25–48)	CONTROL (week 1–24) ACTIVE (week 25–48)
Number	19	24
A1C (%, mmol/mol)	8.32±0.16	8.40±0.15
	67.4±1.75	68.2±1.63
Fasting plasma glucose (mmol/L)	9.5±0.5	10.1±0.5
Body weight (kg)	105.5±4.7	105.8±3.5
Fasting plasma triglycerides (mmol/L)	2.61±0.36	2.41±0.31
Systolic blood pressure (mmHg)	133.3±2.7	136.8±2.7
Diastolic blood pressure (mmHg)	82.2±2.3	87.1±1.7
Plasma cholesterol (mmol/L)	4.62±0.28	5.04±0.27

**Figure 2:**
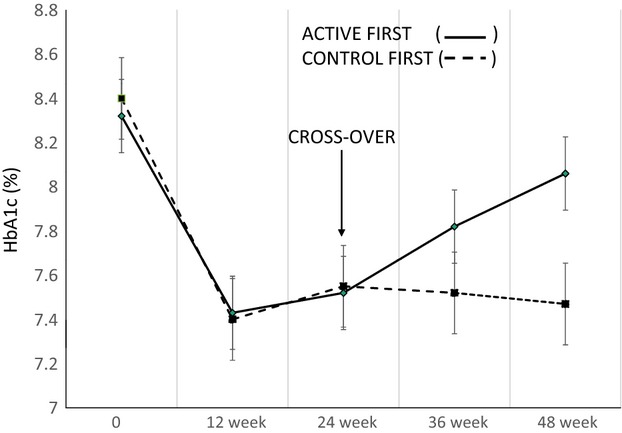
Mean HbA1c throughout period 1 and 2 in both the cohort with electrical stimulation on during period 1 and off during period 2 (solid line) and the cohort with the electrical stimulation off during period 1 and on during period 2 (dashed line). The slope of the solid line is 0.02%/week, *P* = 0.047 and that of the dotted line is −0.002%/week, *P* = 0.78.

Figure2 plots the mean and SEM of HbA1c for both cohorts from baseline through 48 weeks. Surprisingly, the decrease in mean HbA1c in the first period (week 1 through week 24) was similar in both cohorts and was independent of whether the meal-mediated electrical signal was activated or turned off. The maximal decrease in mean HbA1c in both cohorts occurred by 12 weeks and stabilized at 24 weeks. The decrease in HbA1c at 24 weeks was 0.80% (8.8 mmol/mol) in the cohort with the electrical signal activated (ACTIVE) and 0.85% (9.0 mmol/mol) in the cohort with no electrical stimulation (CONTROL). The mean HbA1c at 12 and 24 weeks was statistically significantly decreased from the mean baseline HbA1c value of each cohort (ACTIVE first group *P* = 0.027; CONTROL first group *P* = 0.001).

While the effects in period 1 were similar in both cohorts, the effects in the second period were quite different (Fig.2). When the meal-mediated electrical signal was turned off, there was a steady progressive rise in mean HbA1c from 25 to 48 weeks (slope 0.02%/week, *P* = 0.047) where it reached a mean value of 8.06% (64.2 mmol/mol) which did not differ significantly from the baseline value of 8.32% (67.4 mmol/mol), *P* = 0.465. In contrast, turning on the meal-mediated electrical signal in period 2 maintained the improvement in mean HbA1c throughout the 25th to 48th week (7.47 ± 0.15%; 58.1 ± 1.6 mmol/mol compared to baseline mean HbA1c 8.40 ± 0.15%; 68.2 ± 1.6 mmol/mol, *P* = 0.0011).

**Figure 3:**
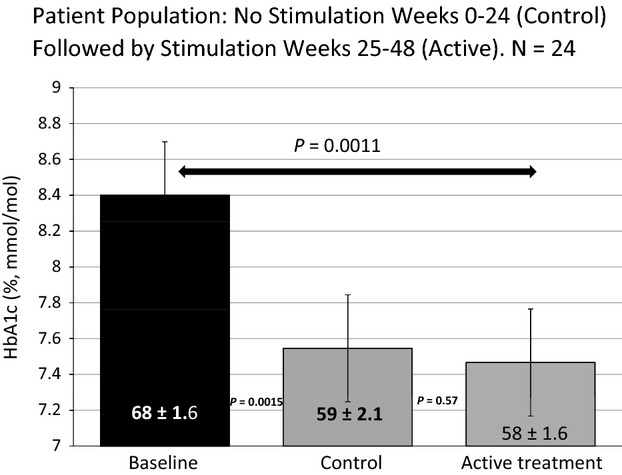
HbA1c data at baseline and at the end of 24 and 48 weeks in patients who started the study with the electrical stimulation off [CONTROL] and had it turned on at 25 weeks.

**Figure 4:**
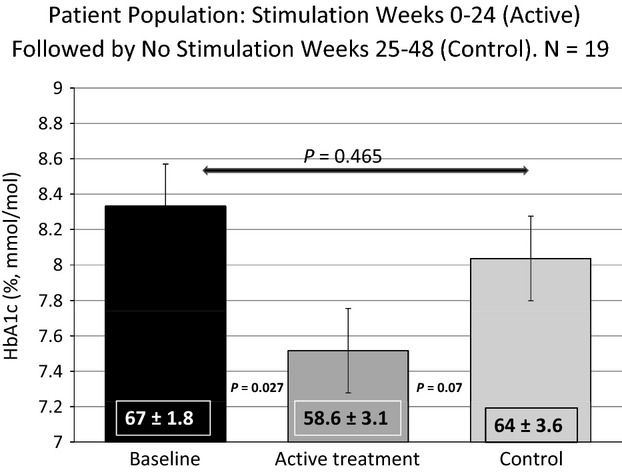
HbA1c data at baseline and 24 and 48 weeks in patients who started with the electrical stimulation on [ACTIVE TREATMENT] and had it turned off at 25 weeks.

The mean ± SE of HbA1c at baseline, and at the end of periods 1 and 2 for each cohort are shown in Figure3 (meal-activated electrical signal off in period 1 and on in period 2) and Figure4 (the meal-activated electrical signal on in period 1 and turned off in period 2).

Implantation of the device itself had an effect in improving HbA1c for the initial 24 weeks independent of the meal-mediated stimulatory signal. The evidence for this device implantation effect are shown in Figures3 and 4. There was a statistically significant decrease in HbA1c in patients implanted with the DIAMOND® without the meal-mediated stimulatory signal being activated (Fig.3 baseline vs. CONTROL), This decrease in mean HbA1c, 7.55 ± 0.19% (59.2 ± 2.1 mmol/mol), was comparable to that occurring in patients implanted with the DIAMOND® with the meal-mediated stimulatory signal activated (Fig.4 baseline vs. ACTIVE TREATMENT) 7.52 ± 0.28% (58.6 ± 3.1 mmol/mol). When the meal-mediated electrical signal was deactivated during the second period (25–48 week), the HbA1c increased to a level that was not statistically significantly different from the baseline HbA1c (mean 8.06 ± 0.32 vs. 8.32 ± 0.16%; 64.2 ± 3.56 vs. 67.4 ± 1.75 mmol/mol) (Fig.4 CONTROL). In contrast, activation of the meal-mediated signal in period 2 (25–48 weeks) in those in whom the signal had not been activated in period 1 maintained improved HbA1c levels equal to the improvement noted in the control period and lower than that in patients with the meal-mediated stimulatory signal inactivated during weeks 25 through 48 (mean 7.47 ± 0.15 vs. 8.06 ± 0.32%; 58.1 ± 1.61 vs. 64.2 ± 3.56 mmol/mol Figure3, ACTIVE TREATMENT. The deterioration in glycemic control during weeks 25 through 48 when the stimulatory signal is disabled indicate that the effects observed from device implantation alone do not persist after 24 weeks (Figs.2, 4, CONTROL).

A formal AB/BA cross-over analysis of the data from the two periods showed no significant carry over effect [*P* = 0.35]. The treatment effect was adjusted for the period effect. The period effects compared the HbA1c measurements between 24 and 48 weeks regardless of the treatment. The period effects were estimated to be −0.22%. *P* = 0.21. The treatment effect [half period] was −0.30, *P* = 0.037 one-sided test. In another analysis, in which the data were analyzed as a parallel trial starting from time 24 weeks and the outcome measured was the difference between HbA1c at 24 and 48 weeks showed the period differences to be −0.60% with a *P* = 0.0374 using the one-sided test.

Measurements of weight, blood pressure, and total cholesterol (Table2) showed a trend toward improvement from baseline through 48 weeks that were greater following period 2 with ACTIVE treatment as compared to CONTROL treatment but these were not significant due to the small numbers of patients in each cohort and the large variability within each cohort.

**Table 2:** Changes in body weight, blood pressure, and plasma cholesterol from baseline to the end of period 1 (24 weeks) and period 2 (48 weeks). The decrease in blood pressure and plasma cholesterol trend to be lower following ACTIVE treatment in period 2 as compared to CONTROL treatment. There was a large variability in weight loss among patients

	Treatment	Baseline	24 weeks	48 weeks	Difference from baseline at 48 weeks
Weight (kg)	Control to Active	105.8±3.5	−2.32±0.94	−2.82±0.88	
	Active to Control	105.5±4.7	−3.35±2.56	−3.39±3.14	
Systolic BP (mmHg)	Control to Active	136.8±2.7	131.8±2.5	133.2±1.9	−3.6
	Active to Control	133.3±2.7	132.7±4.1	135.8±3.1	+2.5
Diastolic BP (mmHg)	Control to Active	87.1±1.7	83.9±1.6	83.7±1.4	−3.4
	Active to Control	82.2±2.3	82.1±2.6	84.9±2.5	+2.7
Plasma cholesterol (mmol/L)	Control to Active	5.04±0.27	4.70±0.26	4.78±0.21	−0.26
	Active to Control	4.62±0.28	4.94±0.25	4.65±0.29	−0.03

## Discussion

The implantation of electrical stimulatory devices can result in two types of mechanistic effects which may influence physiologic responses. The implantation of a device may result in a placebo effect induced by the patient’s perception of a treatment. In adidition, placement of the electrodes themselves may result in alterations in routing of normal impulses and circuits. Either or both of these effects would be independent of the meal-mediated electrical signal and could be either transient or permanent. The second set of changes are those due to the meal-mediated stimulatory electrical signal. This study was designed to determine the extent to which the improvement in metabolic parameters in patients with type 2 diabetes treated with the DIAMOND® gastric electrical stimulatory device is due to the device implantation itself and which effects are due to the chronic meal-mediated signal stimulation. The decrease in HbA1c was the endpoint measured since it is the more rapidly and reproducibly measured metabolic effect of DIAMOND® treatment.

Surprisingly, the data in Figures4 indicate that implantation of the DIAMOND® device itself causes transient alterations, which can be confused with the beneficial effects of the stimulatory electrical signal. This “CONTROL” effect which decreased the HbA1c by approximately 0.85% exists for the first few weeks after implantation, but disappears after 24 weeks. The benefits of the stimulatory electrical signal drive the effects which occur beyond 24 weeks as shown in Figures4. This observation has important implications for devices which involve implanted electrodes. Short-term studies of such devices provide useful safety data but may not be predictive of long-term effects and efficacy.

Several experimental designs have been used to attempt to validate effects due to implantable electrical devices. An implantable gastric stimulator developed by Transneuronix was shown in a prospective, nonrandomized trial with no control group to be associated with a 21 ± 3.5% decrease in excess body weight after 10 months of treatment (De Luca et al. 2004). However, the SHAPE (Screened Health Assessment and Pacer Evaluation) trial with the Transneuronix implantable gastric stimulator for obesity studied parallel implanted populations on a diet with a 500 kcal/day deficit with the device on or off for 1 year and found no difference in weight loss between the groups (mean excess weight loss 11.7% vs. 11.8%) (Shikora et al. 2009). The EMPOWER study which was a randomized, prospective, double-blind multicenter trial of vagal blockade to induce weight loss had two parallel populations: one with the device implanted with leads to the vagal electrodes and one without the leads to the vagal nerve. Body weight loss differences after 12 months were no different (Excess weight loss 17 ± 2% vs. 16 ± 2%) (Sarr et al. 2012). An additional, subsequent 12 month trial (recharge) of vagal blockade in morbidly obese patients reported a 24.4% decrease in excess weight compared to a 15.9% loss in patients with the device implanted but without electrodes attached to the vagus nerve. The study failed to meet its primary endpoint (Ikramuddin et al. 2014). A cross-over design was used in the Multisite Stimulation in Cardiomyopathies (MUSTIC) study to evaluate exercise tolerance, quality of life, peak oxygen uptake, and hospitalizations (Cazeau et al. 2001). Patients with severe heart failure were implanted with the biventricular pacing device and randomized to either 3 months with active pacing or inactive pacing. At the end of the 3 months, the treatments were reversed for the next 3 months. This design indicated that the active periods showed a statistically significant improvement in all the endpoints compared to the inactive periods. The SYMPLICITY HTN trial was a randomized, 6 month prospective, single blind sham-controlled trial of the effect of renal denervation for resistant hypertension (Bhatt et al. 2014). It failed to show any difference in systolic blood pressure, reduction between the groups though both groups showed a significant reduction in systolic blood pressure from baseline. Experience indicates that electrical stimulatory devices must be shown to be effective in appropriate sham-controlled studies.

The importance of randomized, placebo-controlled trials has been validated in many other studies not involving electrical stimulation. A double-blind fluvoxamine/placebo 16 week randomized cross-over trial in pathologic gambling presented results particularly relevant to this study (Hollander et al. 2000). There was no difference in the effect of fluvoxamine compared to placebo in decreasing gambling in the first 8 week period. However, there was a significant benefit of fluvoxamine compared to placebo in reducing gambling in the second 8 week cross-over period. The authors suggested that there was an early placebo effect which diminished over time. Kaptchuk and his colleagues (Kaptchuk et al. 2000) have suggested that placebo effects are not necessarily a baseline measure but may actually be based in physiologic changes and may be important in evaluating medical devices.

A cross-over design has potential advantages over the parallel groups design as each patient is their own control and far fewer patients are required for the study. However, this is only valid if there is either no or a very short-term placebo effect. This unexpectedly turned out not to be true in this study.

The analysis of our cross-over study was complicated by two factors: the long duration of the device implantation effect and the persistence of improved glycemic control for months following intensive normalization of glycemic control. The original analysis plan was to compare the two active treatment periods (Fig.1, periods 2 and 3) to the two control periods (Fig.1, periods 1 and 4). Such an analysis would generate mean decreases in HbA1c in the active treatment periods of 0.88 ± 0.19% (−9.5 ± 2.0 mmol/mol) and in the placebo periods of 0.60 ± 0.20% (−6.44 ± 2.22 mmol/mol), *P* = 0.07. The data in Figures4 show implantation of the device itself transiently alters the endogenous gastric electrical circuitry and causes a significant improvement in glycemic control independent of any external electrical stimulation. This effect is similar to that obtained subsequently with meal-mediated electrical stimulation suggesting that the placebo effect may be transiently activating a similar pathway to that occurring with the meal-mediated electrical signal. Figures2 and 4 show that the device implantation effect is gone by the 25th week as glycemic control in the absence of the antral external electrical stimulation progressively returns toward the baseline HbA1c (8.06 ± 0.32, 64.2 ± 3.56 mmol/mol vs. 8.32 ± 0.16%, 67.4 ± 1.5 mmol/mol, *P* = 0.465) in a nonstimulated state. Extrapolation of the line projects that after an additional 12 weeks the mean value would have achieved the baseline value. For that reason our initial analyses compared mean HbA1c at baseline to those at the ends of periods 1 and 2 in each randomized (ACTIVE TREATMENT first and CONTROL first) group. In the group initiated with ACTIVE TREATMENT, the mean HbA1c in period 1 was statistically significantly reduced compared to baseline and returned to a value not significantly different from baseline when the stimulatory signal was discontinued (Figs.2, 4). When a nonstimulatory period is followed by active stimulation (Figs.2, 3), the second (ACTIVE TREATMENT) period continues to show significant benefit when without stimulation it should have returned to baseline. This is apparent comparing the improvement in period 2 to the baseline mean HbA1c 7.47 ± 0.15% (58.1 ± 1.61 mmol/mol) versus 8.40 ± 0.14% (68.2 ± 1.63%, *P* = 0.0011).

The increase in HbA1c from the ACTIVE TREATMENT to the CONTROL period in weeks 25–48 (0.53 ± 0.27%, 5.58 ± 3.19 mmol/mol) was statistically significantly different (*P* = 0.046) from the decrease in HbA1c from the CONTROL to the ACTIVE TREATMENT period in weeks 25–48 (−0.08 ± 0.15%, −1.08 ± 1.54 mmol/mol). The difference between meal-mediated antral electrical stimulation and no stimulation in improving HbA1c during the second period is further evidence that the chronic effect of the DIAMOND in improving glycemic control is due to the meal-mediated electrical stimulation.

The simple multiple comparison analyses which showed a significant treatment effect of the electrical stimulation is confirmed by the more rigorous AB/BA cross-over analysis with the appropriate Satterthwaite corrections although there is the marked placebo effect in period 1.

This study would be more definitive if we would have been able to add a third randomized period of 24 weeks, however, many patients had significant improvement in their diabetes and most refused to be rerandomized to a third period, which might have had the signal stimulus discontinued. The failure to show a greater difference between the second period stimulus on versus the second period stimulus off (HbA1c difference 0.61%, 6.2 mmol/mol) is explained by the residual benefit of the good glycemic control in period 1 on the second period of the stimulus off such that the mean A1C returned toward (8.06%, 64.2 mmol/mol) but not entirely to the baseline HbA1c level (8.33%, 67.4 mmol/mol). Such prolonged benefit of glycemic control following a period of greatly improved glycemic control has been reported following intensive insulin or oral medication treatment of patients with type 2 diabetes (Banerji et al. 1996; Chen et al. 2008).

The mechanisms by which the implanted device causes temporary improvement in metabolic control are unknown, but the observation that the improvement in glycemic control in the first period was the same whether the electrical signal was activated or not suggest that they may be the same or very similar to those permanently generated by the electrical signal.

In summary, this cross-over study measuring separately the effects of DIAMOND® gastric electrical stimulation device implantation and meal-mediated DIAMOND® antral electrical stimulation show the following:

1. Implantation of the device leads to a significant though transient reduction in HbA1c in patients with type 2 diabetes inadequately controlled with oral agents.
2. The long-term (>24 weeks) improvement in HbA1c is due to the meal-mediated antral electrical stimulation.
3. The effect of the ACTIVE TREATMENT in period 2 with the DIAMOND device in improving glycemic control was a decrease in mean HbA1c from 8.40 ± 0.15% to 7.47 ± 0.15% (−0.93%; −10.1 mmol/mol). This is very similar to the results of a previous open label study in which the decrease in HbA1c in 47 inadequately controlled type 2 diabetic patients after 12 months of DIAMOND treatment was 8.32 ± 0.10% to 7.48 ± 0.15% (−0.84%; −9.5 mmol/mol) (Lebovitz et al. 2015).

## Conflict of Interest

The authors have no additional conflicts of interest.

## Disclosure

Harold E. Lebovitz is a scientific advisor for Metacure, Ltd, Orangeburg, NY, USA and has received travel reimbursement. Bernhard Ludvik, Jaroslaw Kozakowski, and Wieslaw Tarnowski received research grants from Metacure Ltd. Mateusz Zelewski and Irit Yaniv were employees of Metacure Ltd. Tse’ela Schwartz is a statistical consultant for Metacure Ltd. The authors have no additional conflicts of interest.
